# Simulating chalcogen bonding using molecular mechanics: a pseudoatom approach to model ebselen

**DOI:** 10.1007/s00894-021-05023-5

**Published:** 2022-02-24

**Authors:** Thomas Fellowes, Jonathan M. White

**Affiliations:** Bio21 Institute and School of Chemistry, University of Melbourne, Parkville, Australia

**Keywords:** Ebselen, *σ*-hole, Chalcogen bonding, GROMACS, Molecular mechanics.

## Abstract

The organoselenium compound ebselen has recently been investigated as a treatment for COVID-19; however, efforts to model ebselen in silico have been hampered by the lack of an efficient and accurate method to assess its binding to biological macromolecules. We present here a Generalized Amber Force Field modification which incorporates classical parameters for the selenium atom in ebselen, as well as a positively charged pseudoatom to simulate the *σ*-hole, a quantum mechanical phenomenon that dominates the chemistry of ebselen. Our approach is justified using an energy decomposition analysis of a number of density functional theory–optimized structures, which shows that the *σ*-hole interaction is primarily electrostatic in origin. Finally, our model is verified by conducting molecular dynamics simulations on a number of simple complexes, as well as the clinically relevant enzyme SOD1 (superoxide dismutase), which is known to bind to ebselen.

**Electronic supplementary material:**

The online version of this article (10.1007/s00894-021-05023-5) contains supplementary material, which is available to authorized users.

**Graphical Abstract:**
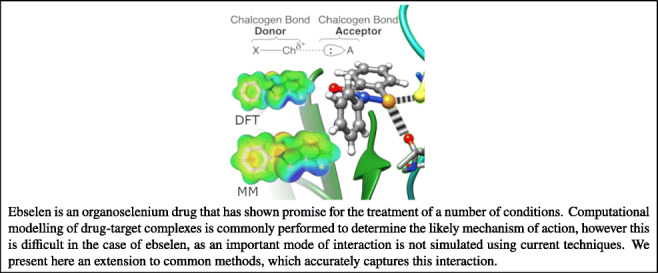
Ebselen is an organoselenium drug that has shown promise for the treatment of a number of conditions. Computational modelling of drug-target complexes is commonly performed to determine the likely mechanism of action, however this is difficult in the case of ebselen, as an important mode of interaction is not simulated using current techniques. We present here an extension to common methods, which accurately captures this interaction.

## Introduction

Ebselen (**1**) is a organoselenium compound that is of great interest to many medicinal chemists, in no small part due its unexpectedly low toxicity for an organoselenium species. Its chemistry is dominated by the highly directional electrophilic nature of the selenium atom, which has not been modelled in force fields in common use. We present here a method to model this chemistry, using a positively charged massless particle on the surface of the selenium atom.

Ebselen was first synthesized in 1924, and its unusual properties went more or less uninvestigated for more than 50 years [1]. Interest in ebselen boomed in the early 1980s due to its antioxidant properties, and since then it has been the subject of several studies into its synthesis, biological properties, and metabolism [2–10]. Its biological activity can be broadly attributed to its ability to neutralize reactive oxygen species (ROS), reducing the level of oxidative stress to which cells are subjected [11]. To this end, ebselen has been investigated for its neuroprotective, mood-stabilizing, anti-inflammatory, and anti-cancer properties [12–17]. Recently it was identified as a compound of interest for the treatment of COVID-19, showing promising inhibition of the viral M^pro^ protease enzyme [18].

The *in vivo* antioxidant ability of ebselen is believed to be mediated through a catalytic cycle analogous to that of glutathione peroxidase (a selenoenzyme) [19]. The selenium-containing heterocycle is reductively opened to afford the free selenol, which is the active catalyst. This is rapidly oxidised by ROS to a selenenic acid, which is then reduced back to the selenol by glutathione (GSH) via a selenenyl sulfide. Its activity against a number of other targets appears to also be mediated through formation of a covalent complex via nucleophilic attack at the selenium. There is also evidence that ebselen interacts with targets non-covalently [18]. These interactions may include association with aromatic or hydrophobic residues, or H-bonding through the carbonyl. Ebselen can also form non-covalent complexes, through the interaction of Lewis bases with an electrophilic *σ*-hole on the selenium atom, similarly to electron-deficient sulfur-containing molecules [20–22].

**Fig. 1:**
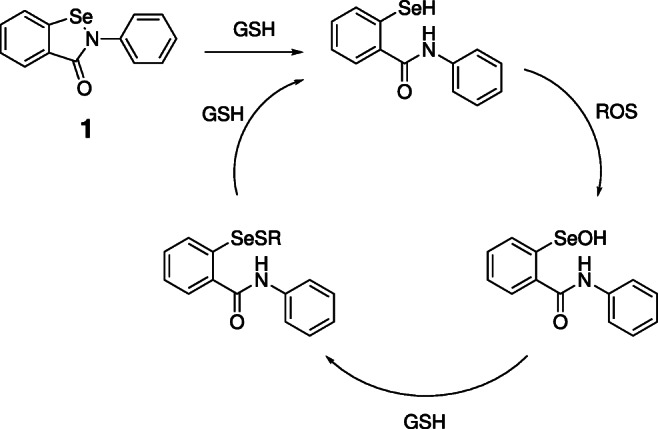
Catalytic cycle of ebselen **1** in vivo

Molecular modelling is a vital tool in drug development, allowing for rapid and broad-reaching screening of drug candidates against likely substrates at minimal cost and risk. Ab initio quantum methods (QM) are widely used to model small molecules (generally smaller than a few hundred atoms), where their accuracy and ability to describe quantum effects underlying photochemical properties and bond breaking/formation processes are critical. They are, however, computationally costly. Molecular mechanics (MM), where systems are treated strictly classically, is a viable alternative for large systems. The drawbacks are that MM relies on having an extensive parameter set (a “force field”) to describe the system (which is not necessarily available, or applicable to the system at hand), and that descriptions of quantum effects often fail (Fig. 1).

Both of these issues are encountered when attempting to model ebselen using MM. Firstly, parameters to describe selenium-containing small molecules are simply not available in most popular force fields, including GAFF, Gromos, and CGenFF. This has been substantially addressed in the work of Torsello et al., where extensive parametrization of a series of diaryl diselenides and diaryl ditellurides was performed, and they provided a methodology to extend this to general chalcogen-containing molecules [23]. They did not, however, address the second issue of quantum effects, which is reasonable given that they do not play a large role in diselenides. The chemistry of ebselen, on the other hand, is dominated by the *σ*-hole, which is a quantum effect [20]. The *σ*-hole is a region of positive electrostatic potential situated opposite to the Se–N bond, which arises due to the strongly anisotropic electron distribution around the selenium atom. This causes the selenium to adopt a highly directional electrophilic character, which can lead to the formation of “chalcogen-bonds” with electron pair donors (named by analogy to the ubiquitous hydrogen bond) [24].

The presence of a *σ*-hole is not a new problem in MM, nor are they exclusive to chalcogens, as they are also found on the heavier halogens, where they give rise to halogen bonding [25]. Perhaps due to the higher prevalence of halogens in drug-like molecules, a number of approaches have been proposed to account for *σ*-holes in halogenated molecules. The common theme in these methods is the inclusion of a pseudoatom with positive electrostatic potential attached to the halogen atom. This pseudoatom is variously called an extra point (EP), explicit *σ*-hole (ESH), or virtual or off-atom centered point, and the approaches differ in the location of the pseudoatom and method used to derive its charge [26–29]. Lone pairs can also be described using negatively charged pseudoatoms, and this approach has been used for some time [29–31].

It is worth noting an alternative approach, used by Cozzolino and Vargas-Baca, which treats secondary bonding interactions as true bonds, with an explicitly parametrized potential [32]. This was used to parametrize the supramolecular synthon 1,2,5-telluradizaole, which is known to self assemble into a range of interesting structures. This approach, however, relies on describing the bond using an *an* harmonic potential, which is not easily implemented in most software.

The inability to model ebselen in biological systems is a major hurdle in understanding the mechanism of its action. Furthermore, recent high-profile work has highlighted the need for an accurate force field which takes quantum effects into account [33, 34]. In this work, we develop a parameter set for the selenium atom in ebselen, including a pseudoatom to simulate the *σ*-hole. We have based the force field on GAFF, due to its popularity and ability to describe most drug like molecules in a way which is compatible with biomolecular force fields. Although GAFF is designed to work in the AMBER molecular dynamics system, we used GROMACS to develop the parameters, as it natively supports massless pseudoatoms which are critical for the description of the *σ*-hole. In principle, the work could be translated to any program that supports massless points, or the pseudoatom could even be given a mass and associated harmonic parameters which would enable some level of polarisability. However, we have simply provided the GROMACS parameters with the massless point for its numerical stability and speed. Using these parameters, we show that this model accurately reproduces experimental geometries and energies, and compares favourably to ab initio calculations. This force field will prove useful in understanding the interactions between ebselen and current targets, and possibly lead to the discovery of new targets.

## Methodology

All quantum calculations were performed using Gaussian16, unless otherwise specified [35]. Electrostatic potentials were calculated using the cubegen program in the Gaussian suite, or mol2cub [36]. The ground state geometry of ebselen was optimized at the *ω* B97M-V/def2TZVP level, followed by vibrational analysis to confirm the structure was minimized [37–39]. The range-separated hybrid *ω* B97M-V has been shown to accurately describe Ch-bonding interactions at modest computational cost [40]. Partial charges were assigned to the atoms using the RESP scheme, at the HF/6-31G* level [41]. This was chosen for consistency with existing AMBER force fields. SAPT(DFT) analyses were conducted using the Psi4 software package on geometries optimized at the *ω* B97M-V/def2TZVP level [42]. Molecular dynamics simulations were performed using GROMACS [43]. The ff14SB force field was used for the SOD1 protein in our validation.

## Results and discussion

We began by deriving the classical bonding parameters involving selenium in ebselen, using the procedure of Torsello [23].

### Classical bonding parameters

Bond and angle force constants were derived by conducting a relaxed potential energy surface scan over a range of ± 0.3 Å for bonds and ± 10^∘^ for angles. The resulting data was truncated to within 5 kcal/mol of the equilibrium energy (at larger distances the surfaces were appreciably anharmonic), and this surface was fitted with a classical harmonic oscillator model (1) using the nls function in the R software package [44]. The equilibrium distance/angle *x*_0_ was fixed to the value from the optimized geometry. The resulting potential energy surfaces and harmonic approximations are shown in fig. S1. Torsion angles were similarly scanned at the DFT and MM (with the torsion term set to zero) levels, and the difference between these surfaces was fitted using a periodic series (2). The resulting parameters are presented in Tables 1 and 2.

1 $$ V(x) = \frac{1}{2} k {(x - x_{0})}^{2}  $$

2 $$ V(\phi) = {\sum}_{n=1} \left( \frac{V_{\max,n}}{2} \times (1 + \cos(n \phi + \gamma_{n})) \right)  $$

**Table 1:** Classical parameters for ebselen. Bond lengths are given in Å, and angles in degrees. Force constants are given in kcal/mol⋅Å^2^ or kcal/mol⋅*radian*^2^

Parameter	*x*_0_	*k*
r(Se-N)	1.8586	434.67
r(Se-C)	1.8829	422.33
$\angle $(C-Se-N)	86.6	610.7
$\angle $(Se-N-C_ar_)	119.6	182.7
$\angle $(Se-N-C_CO_)	115.8	404.5
$\angle $(C-C-Se)	119.4	329.2

**Table 2:** Dihedral parameters for ebselen

Parameter	$V_{\max \limits ,2}$	$V_{\max \limits ,2}$	*γ*_1_	*γ*_2_
	kcal/mol	kcal/mol	^∘^	^∘^
*ϕ*(C_ar_-C_ar_-N-Se)	− 0.9653	0.5108	180	180

Values of 2.12 and 0.2910 for the Lennard-Jones parameters *σ* and *ε* were used for selenium, to account for the polar flattening that is observed in strongly polarised atoms. These values are lower than would be expected for a large atom like selenium (e.g. sulfur’s parameters are only slightly lower at 1.9825 and 0.2824); however, we found that they gave the most realistic bond energies and geometries. This is likely due to errors in the force field which are cancelled out by these artificially low values, which we believe is acceptable seeing as our goal is to provide an internally consistent system rather than absolute truth. The default GAFF Lennard-Jones parameters for the carbonyl oxygen were found to give an unreasonably high barrier to rotation about the central dihedral angle (due to steric repulsion between the oxygen and the aryl hydrogen), so they were changed to 1.5 and 0.08. This did not appear to have any negative effect on the rest of the model.

Default GAFF values were used for all other atoms, and Lorentz/Berthelot mixing rules were used to derive cross-terms.

### Energy decomposition analysis

While attempting to model the *σ*-hole using molecular mechanics, we must remember that we are forcing a classical treatment onto an inherently quantum phenomenon. That said, some parts of the quantum phenomenon are easier than others to treat classically. There are thought to be three attractive energetic components which contribute to a *σ*-hole interaction. Namely, electrostatics, induction, and dispersion [45–47]. The magnitudes of each component of *σ*-hole interactions has been the subject of heated debate in recent years [47–52]. For many applications, these disagreements are fairly philosophical and of little consequence; however, this is not the case when attempting to model *σ*-hole interactions using MM.

The electrostatic component generally refers to the interaction between two static (not distorted by each other) electric fields, which can be graphically represented by visualizing the electrostatic potential surfaces of the donor and acceptor moieties (Fig. 4). This is already treated in MM (for the case of atom centered charges) as a sum of pairwise interactions. The accuracy of this component is only limited by the resolution of the electrostatic potential; it would appear that a pseudoatom approach could thus adequately describe the *σ*-hole. Dispersion is accounted for empirically within the *r*^− 6^ term of the Lennard-Jones potential.

Issues arise when attempting to model the induction component of the *σ*-hole *E*_ind_. This component refers to the redistribution of charge within (polarization) or between (charge-transfer) the donor and acceptor as they approach each other. Movement of charge is simply not accounted for within the most common force fields. This presents a large problem, as charge-transfer drives the strong directionality of *σ*-hole interactions, and may account for a significant proportion of their strength.

To ensure that this is not an insurmountable problem for this parametrization, we conducted energy decomposition analyses (EDA) on a variety of complexes containing ebselen. There are numerous EDA schemes available such as KM-EDA, NEDA, and ALMO; however, we chose to use symmetry-adapted perturbation theory (SAPT) [53, 54]. In contrast to several other schemes, SAPT explicitly includes dispersion (as opposed to adding it as an empirical correction), and contains no physically meaningless “catch-all” energy term. The total interaction energy *E*_tot_ is decomposed into an electrostatic component *E*_elst_, an inductive component *E*_ind_ (this incorporates polarization and charge transfer, as they are not distinct phenomena within the SAPT framework), and a dispersive component *E*_dis_. These attractive forces are balanced by a repulsive exchange component *E*_exch_.

**Fig. 2:**
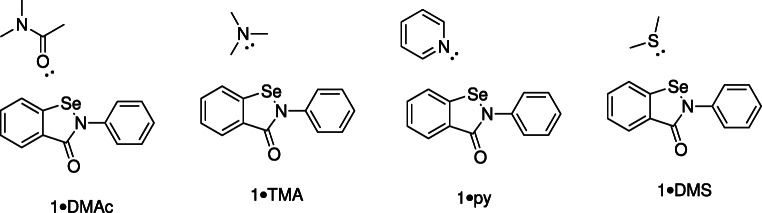
Structures of complexes used for SAPT(DFT) analysis

Four Lewis bases were chosen which are representative of those likely to be encountered in biological systems, and which span a wide range of basicities. Their structures are given in Fig. 2.

**Fig. 3:**
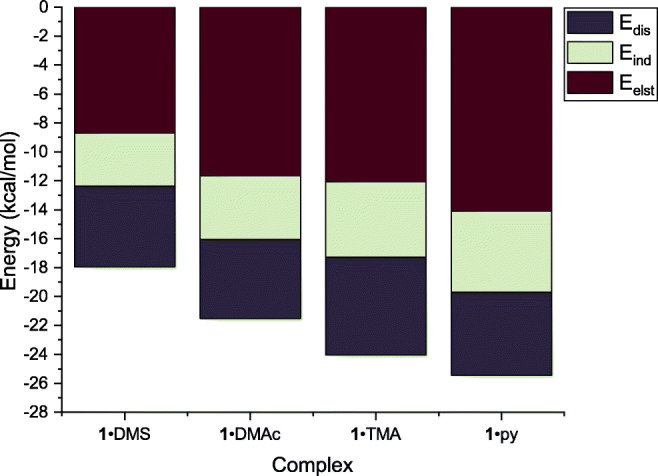
SAPT(DFT) analysis of complexes with four Lewis bases

The SAPT results in Fig. 3 indicate that the majority (at least 70%) of the interaction can be described by electrostatics and dispersion. This suggests that the explicit *σ*-hole parametrization will be reliable, as electrostatics and dispersion are well described by MM.

### Incorporation of pseudoatom

**Fig. 4:**
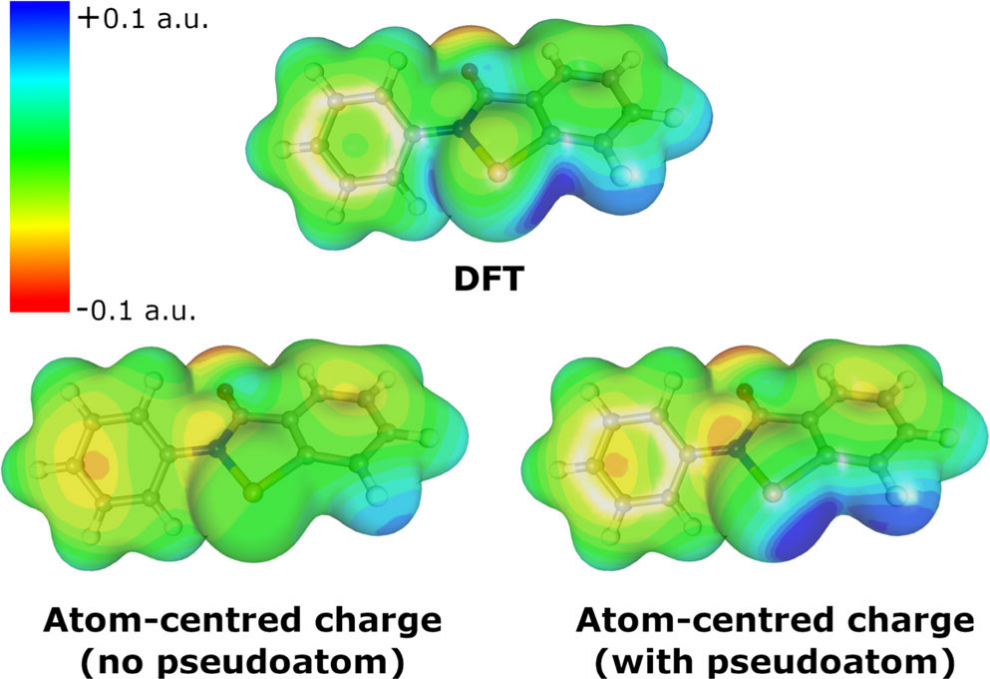
ESP mapped on the 0.005 a.u. electron density isosurface. The *σ*-hole is visible as the dark blue region on the DFT and atom-centered charge (with pseudoatom) surfaces

With classical parameters for ebselen in hand, as well as theoretical assurance that the system can be adequately described using electrostatics, we began to optimize parameters for the pseudoatom representing the *σ*-hole. The pseudoatom was modelled as a virtual site riding on the selenium atom along the extension of the Se-N bond. In GROMACS this is assigned the type 2fd, which is described by only one parameter (the distance along the extension of the defining bond). This is very computationally efficient, although it is an approximation to reality, as the center of the *σ*-hole is actually slightly offset due to the potential from the aromatic ring “spilling” over onto the surface of the selenium (see Fig. 4). The angle $\angle $(N-Se⋯B) (where B is the Lewis basic atom) is also slightly less than 180^∘^, although in the other direction to the true location of the *σ*-hole, an observation which we attribute to sterics. The “off-center” nature of the *σ*-hole has been described before [55]. A more accurate description of the *σ*-hole would be the three-center 3fd or 3fad type; however, this would introduce a performance penalty.

The distance parameter for the pseudoatom was set to 1.189 Å which places the point charge on the VdW surface of the selenium atom. A number of approaches have been suggested to determine this distance; however, it is important for numerical stability of the simulation that the charge lies on or within the VdW surface [28]. The charge of the pseudoatom was calculated using the RESP procedure, which fits a pre-calculated electrostatic potential to atom- (or pseudoatom-) centered point charges while enforcing symmetry restraints. The pseudoatom was then introduced manually, and RESP applied using the ANTECHAMBER program. For comparison, we also calculated charges for the structure *without* a pseudoatom. Relevant charges are presented in Table 3.

**Table 3:** Selected atomic charges for the pseudoatom and no pseudoatom models

Atom	RESP charge (pseudoatom)	RESP charge (no pseudoatom)
E_26_	0.281382	—
Se_1_	− 0.372631	0.056728
N_2_	− 0.241674	− 0.599430
C_3_	0.463064	0.827981
O_4_	− 0.558076	− 0.613468

### Electrostatic potential map

With these parameters in hand, we were able to construct electrostatic potential maps (Fig. 4), which show good qualitative agreement between the DFT and pseudoatom model potentials. Barely visible in the DFT ESP map is a second *σ*-hole, opposite the Se–C bond. Carbon is significantly less electronegative than nitrogen, so it does not polarize the selenium to the same degree, leading to a much smaller *σ*-hole. While it is conceivable that this *σ*-hole could form Ch-bonds as well, we have not observed any evidence of this in any of the derivatives we have studied [21]. We therefore did not attempt to model it, although it could be modelled in the same way as the main *σ*-hole opposite the nitrogen.

### Validation against DFT geometries

A preliminary verification of our model was conducted by comparing the geometries and energies calculated in the SAPT(DFT) analysis with the respective MM values. The Lewis bases chosen for the SAPT(DFT) analysis were constructed in AMBER. GAFF was used for all atoms, and an extra point was added to simulate the lone pair(s) of the Lewis bases per the method of Dixon and Kollman [30]. Geometries were assessed by minimizing the ebselen-Lewis base structure, and energies calculated relative to the unbound minimized monomers. The results are shown in Table 4.

**Table 4:** Median geometric parameters for complexes with **1**

Complex	r(Se⋯B)	$\angle $(N-Se⋯B)	$\angle $(lone pair)	Energy
	Å	^∘^	^∘^	(kcal/mol)
**1**⋅py	2.879 Å (2.775 Å)	174.9^∘^ (176.2^∘^)	171.1^∘^ (165.7^∘^)	− 7.548 (− 7.093)
**1**⋅DMAc	2.741 Å (2.786 Å)	174.7^∘^ (172.4^∘^)	129.1^∘^ (117.9^∘^)	− 7.068 (− 7.551)
**1**⋅TMA	2.928 Å (2.857 Å)	170.1^∘^ (177.4^∘^)	107.2^∘^ (113.6^∘^)	− 5.447 (− 6.627)
**1**⋅DMS	3.253 Å (3.265 Å)	160.4^∘^ (177.4^∘^)	81.2^∘^ (89.8^∘^)	− 2.510 (− 5.646)

DFT equilibrium values are given in brackets for comparison. DFT energies are derived from SAPT(DFT)

These results show that Ch-bonds can be adequately described by the inclusion of a positively charged pseudoatom. Almost all parameters and energies can be adequately reproduced, with the only anomaly being the substantially underestimated bond energy for the **1**⋅DMS complex. Interestingly, the geometry is modelled well in spite of this. This may be an artefact of the parametrization of the sulfur lone pairs [30]. Sulfur compounds are known to be substantially weaker H-bond acceptors than corresponding oxygen compounds, which is expected on the basis of the less negative electrostatic potential on the molecular surface [56]. We believe this may be biasing the results in the case of neutral sulfur-based Ch-bond acceptors.

### Validation against experimental density

We also sought to validate our model against the experimentally determined density of the crystal. An ebselen crystal (CSD code **SENGOH1**, 5 × 5 × 5 unit cells, 1000 molecules) was constructed, and placed in a simulation box of the appropriate size at 1 atm. The crystal was heated to 290 K over 2 ns using a Berendsen thermostat and barostat, and the density was calculated to be 1.548 g/cm^3^, which compares well to the experimental density of 1.529 g/cm^3^ at the same temperature [57]. This procedure was repeated using the non-pseudoatom model, which afforded a density at 290 K of 1.468 g/cm^3^. The pseudoatom clearly represents an improvement when simulating the solid state structure of ebselen.

### Validation against SOD1 binding

**Fig. 5:**
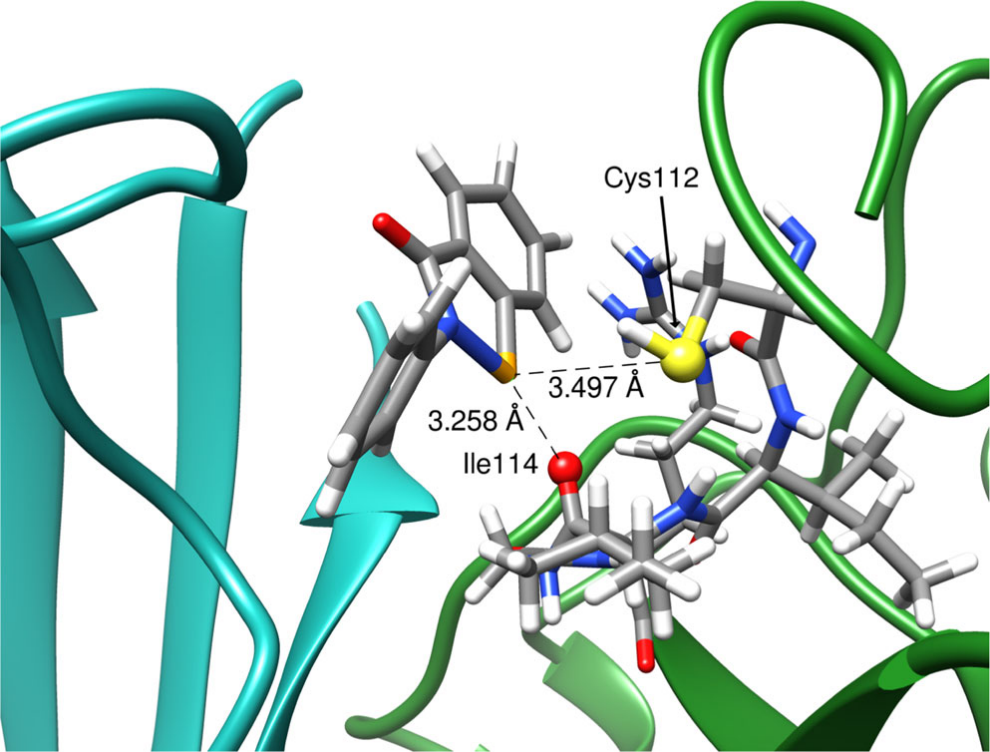
Average binding geometry of ebselen in the SOD1 groove

In order to show the utility of our model in a biological context, we conducted a binding simulation with a known ebselen target. Superoxide dismutase-1 (SOD1) forms a covalent complex with ebselen through the Cys112 residue, which appears to support correct folding of the protein, inhibiting aggregation and associated toxicity [58]. Although formation of the covalent complex cannot be simulated using our model (as this is a bond-forming process), we are able to visualize the stabilized encounter complex which undergoes ring opening to form the final adduct. Indeed, the Ch-bond formed through the *σ*-hole can be thought of as the early stages of a nucleophilic attack at the selenium [20]. SOD1 (PDB **2C9V**) was chosen because of the availability of an atomic resolution structure, demonstrated evidence of ebselen binding, and it’s relatively small size [58, 59]. The structure was prepared by removing disorder, then removing water and ions (the Cu and Zn ions were retained). The ebselen residue was introduced within the binding groove approximately halfway between the two units. The complex was then neutralized by addition of four Na+ ions at the sites of most negative electrostatic potential, and solvated with a TIP3P explicit water model to give a final box size of 77.095 × 96.253 × 78.411 Å. The structure was minimized over 1000 cycles to remove bad contacts, then heated to 300 K over 200 ps. A simulation of 2 ns at 300 K was then performed to assess the average binding geometry, which was found to exhibit a bifurcated Ch-bond between the expected Cys112 sulfur and the adjacent Ile114 backbone carbonyl (Fig. 5). A similar experiment was performed *without* the *σ*-hole, which failed to bind in a reproducible geometry, with the ebselen molecule wandering through the groove. This is presumably driven by hydrophobic interactions, and the entropic cost of desolvation.

## Conclusions

In conclusion, we have developed a set of parameters which can greatly improve modelling of ebselen and its derivatives. Our model gives realistic geometries and energies of gas phase complexes, and reproduces the interaction between ebselen and a protein. Although this work is restricted to ebselen itself, the parameters will be generally applicable to derivatives of ebselen (with appropriate charge fitting). We hope that these results will be useful for the discovery of new targets.

## Electronic supplementary material

Below is the link to the electronic supplementary material.

(PDF 901 KB)
