# Supplementary material for: Simulating chalcogen bonding using molecular mechanics: a pseudoatom approach to model ebselen
Source: J Mol Model. 2022 Feb 24;28(3):66. doi: 10.1007/s00894-021-05023-5 (PMC8867462; doi:10.1007/s00894-021-05023-5)
Supplement: Supplementary file 1 — (PDF 901 KB) [file 894_2021_5023_MOESM1_ESM.pdf]

SI for: Simulating chalcogen bonding using molecular mechanics: A pseudoatom approach to model ebselen.

Thomas Fellowes, Jonathan M. White†

Bio21 Institute and School of Chemistry, University of Melbourne, Parkville, Australia
*fellowes@student.unimelb.edu.au
†whitejm@unimelb.edu.au

August 5, 2021

S1 Potential energy surfaces for harmonic bond approximation

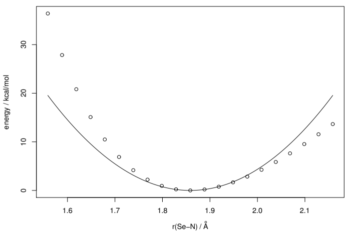

(a) r(Se−N)

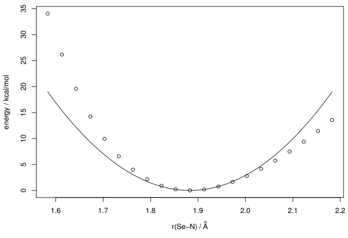

(b) r(Se−C)

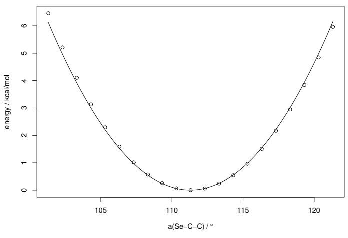

(c) ∠(Se−C_1−C_6)

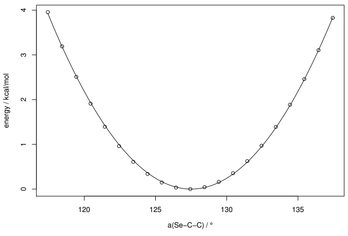

(d) ∠(Se−C_1−C_2)

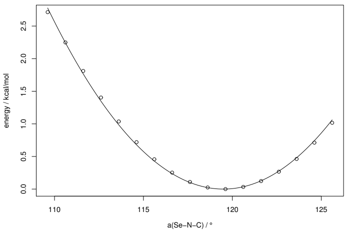

(e) ∠(Se−N−C_8)

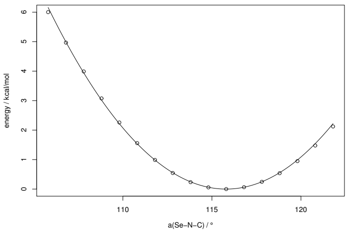

(f) ∠(Se−N−C_7)

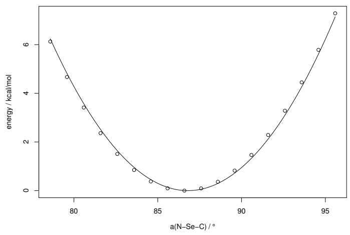

(g) ∠(N−Se−C)

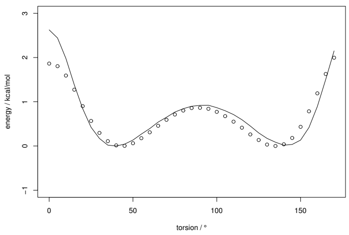

(h) ϕ(Se−N−C−C)

Figure S1: Potential energy surfaces for the indicated geometric parameter. The DFT surface is shown as points and the harmonic approximation is shown as a line.

S2 SAPT(DFT) energy decomposition analysis

Complex	E_{tot}	E_{elst}	E_{ind}	E_{dis}	E_{exch}
**1**·DMAc	-7.551	-11.641	-4.431	-5.447	+13.967
**1**·TMA	-6.627	-12.067	-5.200	-6.777	+17.417
**1**·py	-7.093	-14.093	-5.605	-5.750	+18.358
**1**·DMS	-5.646	-8.703	-3.669	-5.571	+12.297

Table S1: SAPT(DFT) analysis of complexes with four Lewis bases. All energies are given in kcal/mol.

Atom	x	y	z
Se1	0.1103	-0.3014	-0.026
O2	-3.0289	2.1045	-0.0599
N3	-1.6949	0.2302	-0.0513
C4	0.5087	1.5474	-0.0469
C5	1.7598	2.1522	-0.0498
H6	2.6615	1.5581	-0.038
C7	-0.6467	2.3169	-0.0605
C8	-1.9311	1.5839	-0.0541
C9	1.8252	3.5343	-0.0666
H10	2.7953	4.0158	-0.0681
C11	-2.7064	-0.7562	-0.0295
C12	0.6692	4.3127	-0.082
H13	0.7454	5.392	-0.096
C14	-0.5697	3.7033	-0.0792
H15	-1.4905	4.2727	-0.0913
C16	-3.8504	-0.5892	0.7469
H17	-3.9822	0.3191	1.3145
C18	-2.5475	-1.9213	-0.7739
H19	-1.6709	-2.0451	-1.3977
C20	-3.5143	-2.9128	-0.7341
H21	-3.3765	-3.8145	-1.3174
C22	-4.8167	-1.58	0.7676
H23	-5.7049	-1.4384	1.3704
C24	-4.6546	-2.7463	0.0343
N25	2.8164	-0.9091	0.0522
C26	3.3736	-1.2146	1.2201
C27	3.4725	-1.2228	-1.0609
C28	4.6048	-1.8402	1.3265
H29	2.805	-0.946	2.1039
C30	4.7079	-1.8489	-1.0562
H31	2.9832	-0.9598	-1.9926
C32	5.2842	-2.1631	0.1635
H33	5.0165	-2.068	2.3003
H34	5.2022	-2.0836	-1.9891
H35	-5.4133	-3.5177	0.0602
H36	6.2485	-2.6533	0.2071

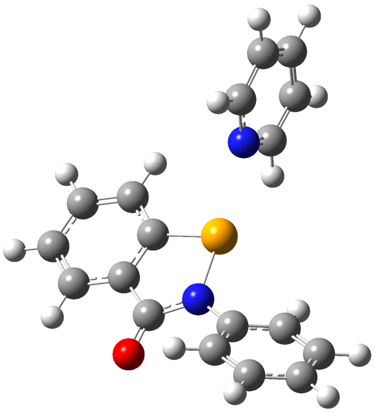

Table S2: **1**·py coordinates. Total energy = -3280.424888 a.u.

Atom	x	y	z
Se1	0.0054	-0.1461	-0.4289
O2	-3.3159	1.8035	0.4379
N3	-1.8121	0.1369	-0.0642
C4	0.1873	1.7264	-0.2918
C5	1.341	2.4794	-0.4723
H6	2.2729	1.9967	-0.7306
C7	-1.0211	2.3349	0.0175
C8	-2.189	1.4409	0.1658
C9	1.2554	3.8524	-0.3223
H10	2.1451	4.4544	-0.4611
C11	-2.685	-0.9725	0.002
C12	0.0474	4.4724	-0.0046
H13	0.0064	5.5483	0.1047
C14	-1.0945	3.7142	0.1628
H15	-2.0511	4.1612	0.4021
C16	-3.6716	-1.0489	0.9819
H17	-3.7934	-0.2361	1.6813
C18	-2.5416	-2.014	-0.91
H19	-1.7914	-1.9472	-1.6884
C20	-3.3672	-3.124	-0.8373
H21	-3.2441	-3.9266	-1.5534
C22	-4.5001	-2.1565	1.036
H23	-5.2679	-2.2044	1.798
C24	-4.3521	-3.1998	0.1339
H25	-5.0022	-4.0636	0.1862
C26	3.4197	-0.7051	0.2768
O27	2.773	-0.3286	-0.6957
N28	4.3777	-1.6549	0.1587
C29	4.671	-2.2259	-1.1397
H30	5.7129	-2.0334	-1.4096
H31	4.5127	-3.3072	-1.12
H32	4.0172	-1.7786	-1.8813
C33	5.1973	-2.1448	1.2436
H34	5.161	-3.2368	1.2689
H35	6.2405	-1.8434	1.1077
H36	4.8535	-1.775	2.2036
C37	3.1632	-0.116	1.6452
H38	2.7896	-0.8732	2.3361
H39	4.0674	0.3202	2.0716
H40	2.4094	0.6593	1.5426

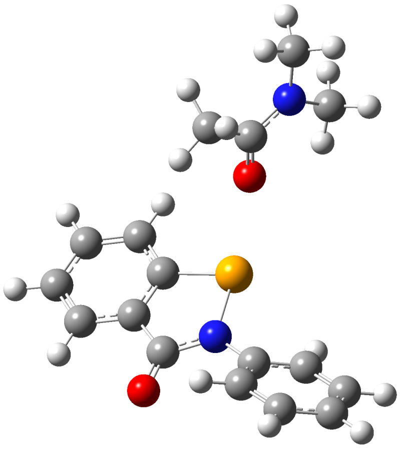

Table S3: **1**·DMAc coordinates. Total energy = -3319.951828 a.u.

Atom	x	y	z
Se1	0.3271	-0.5347	-0.0229
O2	-1.8488	2.7657	-0.0742
N3	-1.2023	0.5583	-0.063
C4	1.3072	1.0775	-0.0525
C5	2.6867	1.2317	-0.0591
H6	3.34	0.3727	-0.0495
C7	0.4723	2.1859	-0.0708
C8	-0.9819	1.9154	-0.0661
C9	3.2102	2.5121	-0.0811
H10	4.285	2.6441	-0.0858
C11	-2.4807	-0.0439	-0.0415
C12	2.3777	3.6298	-0.099
H13	2.8082	4.6223	-0.1172
C14	1.0063	3.4674	-0.0948
H15	0.328	4.3112	-0.1105
C16	-3.5014	0.4766	0.7494
H17	-3.3249	1.3696	1.3294
C18	-2.7151	-1.186	-0.8012
H19	-1.9309	-1.5783	-1.437
C20	-3.9523	-1.8083	-0.762
H21	-4.1213	-2.6966	-1.3574
C22	-4.7382	-0.1445	0.7694
H23	-5.5277	0.2705	1.3832
C24	-4.9703	-1.2893	0.0211
H25	-5.9392	-1.7709	0.0465
C26	3.4784	-2.2979	-1.0177
H27	4.1383	-3.1802	-1.0084
H28	2.8857	-2.3182	-1.9329
H29	4.1074	-1.4073	-1.0404
C30	1.7472	-3.4411	0.1639
H31	1.1614	-3.4979	-0.7548
H32	2.3328	-4.3686	0.2596
H33	1.0574	-3.3811	1.007
C34	3.3391	-2.1369	1.3768
H35	3.9965	-3.0025	1.5567
H36	3.958	-1.2393	1.3564
H37	2.6435	-2.05	2.2121
N38	2.5966	-2.2636	0.1352

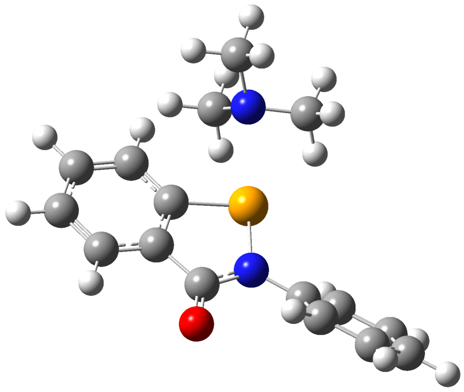

Table S4: **1**·TMA coordinates. Total energy = -3206.593859 a.u.

Atom	x	y	z
Se1	0.3065	-0.5154	-0.4
O2	-1.6865	2.7999	0.3621
N3	-1.1594	0.6101	-0.0963
C4	1.3641	1.0435	-0.2832
C5	2.7386	1.1523	-0.4498
H6	3.3403	0.2878	-0.6942
C7	0.5897	2.1611	-0.0082
C8	-0.867	1.9387	0.1184
C9	3.3194	2.4011	-0.3155
H10	4.3899	2.5017	-0.4436
C11	-2.4564	0.0507	-0.0281
C12	2.5504	3.5276	-0.0274
H13	3.027	4.494	0.0711
C14	1.1825	3.4101	0.122
H15	0.5525	4.2644	0.3353
C16	-3.3589	0.4607	0.9492
H17	-3.0781	1.2378	1.6436
C18	-2.8253	-0.9381	-0.9348
H19	-2.134	-1.2402	-1.7121
C20	-4.0801	-1.5202	-0.8584
H21	-4.355	-2.2887	-1.5696
C22	-4.6154	-0.1173	1.0065
H23	-5.3136	0.2114	1.7658
C24	-4.9816	-1.1109	0.1104
H25	-5.9647	-1.5601	0.1655
S26	2.9125	-2.4561	-0.0815
C27	3.0637	-1.9613	1.6424
H28	3.7192	-1.0921	1.6749
H29	2.0898	-1.6829	2.0461
H30	3.5034	-2.7613	2.2375
C31	1.7527	-3.8193	0.0936
H32	1.5578	-4.2111	-0.9034
H33	2.1795	-4.6108	0.709
H34	0.8151	-3.4741	0.5323

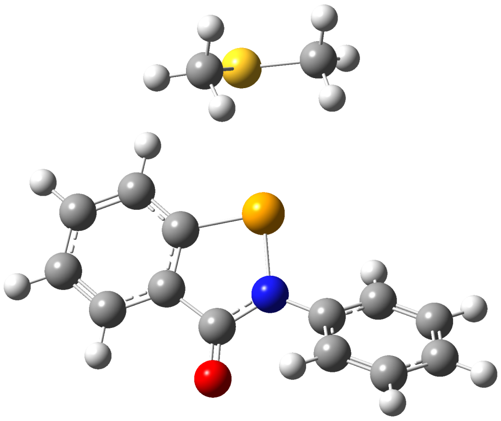

Table S5: **1**·DMS coordinates. Total energy =-3510.182468 a.u.

S3 .itp file for ebselen

```
[ moleculetype ]
; Name            nrexcl
EBS          3

[ atoms ]
;   nr       type  resnr residue  atom   cgnr    charge       mass  typeB    chargeB      massB
; residue    1 EBS rtp EBS q -0.0
    1         se      1    EBS    Se1      1 -0.37263100  78.960000   ; qtot -0.372631
    2          n      1    EBS     N2      2 -0.24167400  14.010000   ; qtot -0.614305
    3          c      1    EBS     C3      3 0.46306400  12.010000   ; qtot -0.151241
    4          o      1    EBS     O4      4 -0.55807600  16.000000   ; qtot -0.709317
    5         ca      1    EBS     C5      5 -0.12908300  12.010000   ; qtot -0.838400
    6         ca      1    EBS     C6      6 0.31112400  12.010000   ; qtot -0.527276
    7         ca      1    EBS     C7      7 -0.34240000  12.010000   ; qtot -0.869676
    8         ca      1    EBS     C8      8 -0.02145900  12.010000   ; qtot -0.891135
    9         ca      1    EBS     C9      9 -0.23294300  12.010000   ; qtot -1.124078
   10         ca      1    EBS    C10     10 -0.08660600  12.010000   ; qtot -1.210684
   11         ca      1    EBS    C11     11 0.31005300  12.010000   ; qtot -0.900631
   12         ca      1    EBS    C12     12 -0.12767700  12.010000   ; qtot -1.028308
   13         ca      1    EBS    C13     13 -0.22332900  12.010000   ; qtot -1.251637
   14         ca      1    EBS    C14     14 -0.11624900  12.010000   ; qtot -1.367886
   15         ca      1    EBS    C15     15 -0.22332900  12.010000   ; qtot -1.591215
   16         ca      1    EBS    C16     16 -0.12767700  12.010000   ; qtot -1.718892
   17         ha      1    EBS    H17     17 0.19878600   1.008000   ; qtot -1.520106
   18         ha      1    EBS    H18     18 0.13050400   1.008000   ; qtot -1.389602
   19         ha      1    EBS    H19     19 0.16889500   1.008000   ; qtot -1.220707
   20         ha      1    EBS    H20     20 0.16852300   1.008000   ; qtot -1.052184
   21         ha      1    EBS    H21     21 0.14768400   1.008000   ; qtot -0.904500
   22         ha      1    EBS    H22     22 0.16570000   1.008000   ; qtot -0.738800
   23         ha      1    EBS    H23     23 0.14403200   1.008000   ; qtot -0.594768
   24         ha      1    EBS    H24     24 0.16570000   1.008000   ; qtot -0.429068
   25         ha      1    EBS    H25     25 0.14768400   1.008000   ; qtot -0.281384
   26         lp      1    EBS    E26     26 0.28138200   0.000000   ; qtot -0.000002

   [ virtual_sites2 ]
; Site   from        funct  d
  26     1     2     2      -0.1189

[ bonds ]
;    ai     aj funct         c0         c1         c2         c3
      1      2     1    0.18586 363731.856000
      1      5     1    0.18829 353405.744000
      1     26     1    0.10600 173887.040000
      2      3     1    0.13789 357815.680000
      2     11     1    0.14121 321498.560000
      3      4     1    0.12183 533627.360000
      3      6     1    0.14906 289449.120000
```

```
         5        6       1    0.13984 385848.480000
         5       10       1    0.13984 385848.480000
         6        7       1    0.13984 385848.480000
         7        8       1    0.13984 385848.480000
         8        9       1    0.13984 385848.480000
         9       10       1    0.13984 385848.480000
        11       12       1    0.13984 385848.480000
        11       16       1    0.13984 385848.480000
        12       13       1    0.13984 385848.480000
        13       14       1    0.13984 385848.480000
        14       15       1    0.13984 385848.480000
        15       16       1    0.13984 385848.480000
         7       17       1    0.10860 289365.440000
         8       18       1    0.10860 289365.440000
         9       19       1    0.10860 289365.440000
        10       20       1    0.10860 289365.440000
        12       21       1    0.10860 289365.440000
        13       22       1    0.10860 289365.440000
        14       23       1    0.10860 289365.440000
        15       24       1    0.10860 289365.440000
        16       25       1    0.10860 289365.440000

[ pairs ]
;    ai      aj funct          c0          c1          c2          c3
         1        4       1
         1       12       1
         1       16       1
         1        7       1
         1        9       1
         2       10       1
         2        7       1
         2       13       1
         2       15       1
        26        3       1
         3       12       1
         3       16       1
         3       10       1
         3        8       1
         4       11       1
         4        5       1
         4        7       1
         5       11       1
         5        8       1
         6       11       1
        26        6       1
         6        9       1
         7       10       1
        26       10       1
        26       11       1
```

```
    11    14     1
    12    15     1
    13    16     1
     1    20     1
     2    21     1
     2    25     1
     3    17     1
     5    17     1
     5    19     1
     6    20     1
     6    18     1
     7    19     1
     8    20     1
     9    17     1
    10    18     1
    11    22     1
    11    24     1
    12    25     1
    12    23     1
    13    24     1
    14    21     1
    14    25     1
    15    22     1
    16    21     1
    16    23     1
    17    18     1
    18    19     1
    19    20     1
    21    22     1
    22    23     1
    23    24     1
    24    25     1

[ angles ]
;    ai    aj    ak funct        c0         c1         c2         c3
     1     2     3     1   115.8000496 3384.856000
     1     2    11     1   119.6000513 1528.833600
     1     5     6     1   119.4000512 2754.745600
     1     5    10     1   119.4000512 2754.745600
     2     1     5     1   86.6000371 5110.337600
     2     1    26     1   180.0000771 418.400000
     2     3     4     1   123.0500530 620.905600
     2     3     6     1   115.2500497 566.513600
     2    11    12     1   120.1900517 568.187200
     2    11    16     1   120.1900517 568.187200
     3     2    11     1   123.7100528 533.878400
     3     6     5     1   120.3300516 538.062400
     3     6     7     1   120.3300516 538.062400
     4     3     6     1   122.6000525 574.881600
```

```
      5      1     26     1   93.4000400 418.400000
      5      6      7     1   120.0200517 557.308800
      5     10      9     1   120.0200517 557.308800
      6      5     10     1   120.0200517 557.308800
      6      7      8     1   120.0200517 557.308800
      7      8      9     1   120.0200517 557.308800
      8      9     10     1   120.0200517 557.308800
     11     12     13     1   120.0200517 557.308800
     11     16     15     1   120.0200517 557.308800
     12     11     16     1   120.0200517 557.308800
     12     13     14     1   120.0200517 557.308800
     13     14     15     1   120.0200517 557.308800
     14     15     16     1   120.0200517 557.308800
      5     10     20     1   119.8800511 403.337600
      6      7     17     1   119.8800511 403.337600
      7      8     18     1   119.8800511 403.337600
      8      7     17     1   119.8800511 403.337600
      8      9     19     1   119.8800511 403.337600
      9      8     18     1   119.8800511 403.337600
      9     10     20     1   119.8800511 403.337600
     10      9     19     1   119.8800511 403.337600
     11     12     21     1   119.8800511 403.337600
     11     16     25     1   119.8800511 403.337600
     12     13     22     1   119.8800511 403.337600
     13     12     21     1   119.8800511 403.337600
     13     14     23     1   119.8800511 403.337600
     14     13     22     1   119.8800511 403.337600
     14     15     24     1   119.8800511 403.337600
     15     14     23     1   119.8800511 403.337600
     15     16     25     1   119.8800511 403.337600
     16     15     24     1   119.8800511 403.337600

[ dihedrals ]
;    ai     aj     ak     al funct         c0          c1          c2          c3          c4          c5
      1      2      3      4     1  180.0000771  10.4600000  2
      1      2      3      6     1  180.0000771  10.4600000  2
      1      2     11     12     1  180.0000771  -4.0388152  1
      1      2     11     12     1  180.0000771  2.1371872  2
      1      2     11     16     1  180.0000771  -4.0388152  1
      1      2     11     16     1  180.0000771  2.1371872  2
      1      5      6      3     1  180.0000771  15.1670000  2
      1      5      6      7     1  180.0000771  15.1670000  2
      1      5     10      9     1  180.0000771  15.1670000  2
      2      1      5      6     1  180.0000771  -5.2103352  1
      2      1      5      6     1  180.0000771  0.5167240  2
      2      1      5      6     1  180.0000771  -5.9860488  3
      2      1      5      6     1  180.0000771  0.6773896  4
      2      1      5     10     1  180.0000771  -5.2103352  1
      2      1      5     10     1  180.0000771  0.5167240  2
```

2	1	5	10	1	180.0000771	-5.9860488	3
2	1	5	10	1	180.0000771	0.6773896	4
2	3	6	5	1	180.0000771	4.1840000	2
2	3	6	7	1	180.0000771	4.1840000	2
2	11	12	13	1	180.0000771	15.1670000	2
2	11	16	15	1	180.0000771	15.1670000	2
5	1	2	3	1	180.0000771	-5.2103352	1
5	1	2	3	1	180.0000771	0.5167240	2
5	1	2	3	1	180.0000771	-5.9860488	3
5	1	2	3	1	180.0000771	0.6773896	4
26	1	2	3	1	180.0000771	-5.2103352	1
26	1	2	3	1	180.0000771	0.5167240	2
26	1	2	3	1	180.0000771	-5.9860488	3
26	1	2	3	1	180.0000771	0.6773896	4
3	2	11	12	1	180.0000771	0.0000000	1
3	2	11	16	1	180.0000771	0.0000000	1
3	6	5	10	1	180.0000771	15.1670000	2
3	6	7	8	1	180.0000771	15.1670000	2
4	3	2	11	1	180.0000771	10.4600000	2
4	3	6	5	1	180.0000771	4.1840000	2
4	3	6	7	1	180.0000771	4.1840000	2
5	1	2	11	1	180.0000771	-5.2103352	1
5	1	2	11	1	180.0000771	0.5167240	2
5	1	2	11	1	180.0000771	-5.9860488	3
5	1	2	11	1	180.0000771	0.6773896	4
5	6	7	8	1	180.0000771	15.1670000	2
5	10	9	8	1	180.0000771	15.1670000	2
6	3	2	11	1	180.0000771	10.4600000	2
26	1	5	6	1	180.0000771	-5.2103352	1
26	1	5	6	1	180.0000771	0.5167240	2
26	1	5	6	1	180.0000771	-5.9860488	3
26	1	5	6	1	180.0000771	0.6773896	4
6	5	10	9	1	180.0000771	15.1670000	2
6	7	8	9	1	180.0000771	15.1670000	2
7	6	5	10	1	180.0000771	15.1670000	2
7	8	9	10	1	180.0000771	15.1670000	2
26	1	5	10	1	180.0000771	-5.2103352	1
26	1	5	10	1	180.0000771	0.5167240	2
26	1	5	10	1	180.0000771	-5.9860488	3
26	1	5	10	1	180.0000771	0.6773896	4
26	1	2	11	1	180.0000771	-5.2103352	1
26	1	2	11	1	180.0000771	0.5167240	2
26	1	2	11	1	180.0000771	-5.9860488	3
26	1	2	11	1	180.0000771	0.6773896	4
11	12	13	14	1	180.0000771	15.1670000	2
11	16	15	14	1	180.0000771	15.1670000	2
12	11	16	15	1	180.0000771	15.1670000	2
12	13	14	15	1	180.0000771	15.1670000	2
13	12	11	16	1	180.0000771	15.1670000	2

```
   13    14    15    16    1  180.0000771  15.1670000  2
    1     2    11     3    4  180.0000771  4.6024000  2
    6     2     3     4    4  180.0000771  4.6024000  2
    1     5    10     6    4  180.0000771  4.6024000  2
    3     5     6     7    4  180.0000771  4.6024000  2
   12    16    11     2    4  180.0000771  4.6024000  2
    1     5    10    20    1  180.0000771  15.1670000  2
    2    11    12    21    1  180.0000771  15.1670000  2
    2    11    16    25    1  180.0000771  15.1670000  2
    3     6     7    17    1  180.0000771  15.1670000  2
    5     6     7    17    1  180.0000771  15.1670000  2
    5    10     9    19    1  180.0000771  15.1670000  2
    6     5    10    20    1  180.0000771  15.1670000  2
    6     7     8    18    1  180.0000771  15.1670000  2
    7     8     9    19    1  180.0000771  15.1670000  2
    8     9    10    20    1  180.0000771  15.1670000  2
    9     8     7    17    1  180.0000771  15.1670000  2
   10     9     8    18    1  180.0000771  15.1670000  2
   11    12    13    22    1  180.0000771  15.1670000  2
   11    16    15    24    1  180.0000771  15.1670000  2
   12    11    16    25    1  180.0000771  15.1670000  2
   12    13    14    23    1  180.0000771  15.1670000  2
   13    14    15    24    1  180.0000771  15.1670000  2
   14    13    12    21    1  180.0000771  15.1670000  2
   14    15    16    25    1  180.0000771  15.1670000  2
   15    14    13    22    1  180.0000771  15.1670000  2
   16    11    12    21    1  180.0000771  15.1670000  2
   16    15    14    23    1  180.0000771  15.1670000  2
   17     7     8    18    1  180.0000771  15.1670000  2
   18     8     9    19    1  180.0000771  15.1670000  2
   19     9    10    20    1  180.0000771  15.1670000  2
   21    12    13    22    1  180.0000771  15.1670000  2
   22    13    14    23    1  180.0000771  15.1670000  2
   23    14    15    24    1  180.0000771  15.1670000  2
   24    15    16    25    1  180.0000771  15.1670000  2
    6     8     7    17    4  180.0000771  4.6024000  2
    7     9     8    18    4  180.0000771  4.6024000  2
    8    10     9    19    4  180.0000771  4.6024000  2
    5     9    10    20    4  180.0000771  4.6024000  2
   11    13    12    21    4  180.0000771  4.6024000  2
   12    14    13    22    4  180.0000771  4.6024000  2
   13    15    14    23    4  180.0000771  4.6024000  2
   14    16    15    24    4  180.0000771  4.6024000  2
   11    15    16    25    4  180.0000771  4.6024000  2

[ system ]
; Name
Ebselen
```
